# Activation of *Akt* Signaling Reduces the Prevalence and Intensity of Malaria Parasite Infection and Lifespan in *Anopheles stephensi* Mosquitoes

**DOI:** 10.1371/journal.ppat.1001003

**Published:** 2010-07-15

**Authors:** Vanessa Corby-Harris, Anna Drexler, Laurel Watkins de Jong, Yevgeniya Antonova, Nazzy Pakpour, Rolf Ziegler, Frank Ramberg, Edwin E. Lewis, Jessica M. Brown, Shirley Luckhart, Michael A. Riehle

**Affiliations:** 1 Department of Entomology, University of Arizona, Tucson, Arizona, United States of America; 2 Medical Microbiology and Immunology, University of California – Davis, Davis, California, United States of America; 3 Department of Entomology, University of California – Davis, Davis, California, United States of America; 4 Department of Nematology, University of California – Davis, Davis, California, United States of America; Institut Pasteur, France

## Abstract

Malaria (*Plasmodium* spp.) kills nearly one million people annually and this number will likely increase as drug and insecticide resistance reduces the effectiveness of current control strategies. The most important human malaria parasite, *Plasmodium falciparum*, undergoes a complex developmental cycle in the mosquito that takes approximately two weeks and begins with the invasion of the mosquito midgut. Here, we demonstrate that increased Akt signaling in the mosquito midgut disrupts parasite development and concurrently reduces the duration that mosquitoes are infective to humans. Specifically, we found that increased Akt signaling in the midgut of heterozygous *Anopheles stephensi* reduced the number of infected mosquitoes by 60–99%. Of those mosquitoes that were infected, we observed a 75–99% reduction in parasite load. In homozygous mosquitoes with increased Akt signaling parasite infection was completely blocked. The increase in midgut-specific Akt signaling also led to an 18–20% reduction in the average mosquito lifespan. Thus, activation of Akt signaling reduced the number of infected mosquitoes, the number of malaria parasites per infected mosquito, and the duration of mosquito infectivity.

## Author Summary

For malaria transmission to occur, a mosquito must ingest and harbor the parasites for approximately two weeks while the parasites complete multiple developmental stages. Until development is complete and the malaria parasites invade the mosquito salivary glands, transmission to another host cannot occur. Upon completion of parasite development, transmission is possible with every subsequent bite. In this study we demonstrate that tissue-specific overexpression of a single activated protein kinase that is essential to insulin signaling in the mosquito can dramatically reduce parasite development. This kinase – Akt – has been described as a critical cell signaling node that regulates a range of physiological processes. In addition to the impact on parasite development, increased Akt signaling also reduced the average mosquito lifespan relative to controls, thereby limiting the window of opportunity for successful parasite transmission. Thus, we demonstrate that genetic manipulation of one key signaling protein directly reduces parasite development in the insect vector as well as the duration of mosquito infectivity.

## Introduction

Malaria is one of the world's most severe public health concerns, killing nearly one million people annually [1]. The disease is caused by infection with parasites of the genus *Plasmodium* that are transmitted by female anopheline mosquitoes. Shortly after an infective bloodmeal is consumed by the mosquito, motile ookinetes develop and attempt to invade the mosquito midgut. Ookinetes that successfully traverse the midgut epithelium form non-motile oocysts and develop on the midgut for a minimum of 12 days before rupturing and releasing sporozoites capable of invading the salivary glands. Following salivary gland invasion by sporozoites, and within 16 days after ingestion of an infectious bloodmeal, the mosquito becomes infective to humans and remains so for the duration of its life. Midgut invasion by the parasite is highly risky and a majority of the parasites perish before developing into oocysts [2], [3]. Further, *Anopheles stephensi* mosquitoes – the leading vector of malaria in India, parts of Asia and the Middle East and the focus of our work – rarely survive more than two weeks in the field [4]–[6]. These observations suggest that only the oldest mosquitoes in a population are capable of transmitting malaria and that even a modest reduction in lifespan could significantly impact parasite transmission.

The insulin/insulin-like growth factor 1 signaling (IIS) cascade plays a critical role in the regulation of innate immunity and lifespan in a wide range of vertebrate and invertebrate organisms [7], [8]. IIS is initiated through the binding of insulin-like peptides (ILPs) to the insulin receptor, leading to a series of downstream phosphorylation events that include the key signaling protein Akt. Activation of IIS results in translocation of Akt to the cell membrane where it is phosphorylated and activated by phosphoinositide-dependent kinase-1 (PDK1). Activated Akt then phosphorylates the forkhead transcription factor FOXO1, preventing it from entering the nucleus and activating transcription of target genes [9].

In model invertebrates, the IIS cascade has been linked to both innate immunity and lifespan regulation. In the nematode *Caenorhabditis elegans*, disruption of the insulin receptor orthologue *daf-2* leads to decreased IIS, extension of lifespan [10] and increased resistance to bacterial infection [11]. In contrast, loss of function mutations in the FOXO1 orthologue *daf-16* result in nematodes that are sensitive to infection [11] and short-lived [12]. As in *C. elegans*, disruption of the IIS can lead to lifespan extension in the fruit fly *Drosophila melanogaster* [13]–[15]. Recent work has also demonstrated that activation of the Toll cascade, a key pathway in fly immunity, inhibits IIS in the fly [16]. These observations confirm that the connections observed in humans between innate immunity, metabolism and aging are evolutionarily conserved (reviewed in [17]).

Lifespan extension due to IIS disruption is tissue-dependant, although the tissues involved can vary within and across genera. In *C. elegans* [18] and *D. melanogaster* [15], the nervous system is a key IIS center. In *D. melanogaster*, disruption of IIS in the fat body can also lead to lifespan extension [15]. Overexpression of the transcription factor daf-16 in the *C. elegans* intestine extends lifespan [19]. Our previous work with *A. stephensi* suggests that the analogous mosquito tissue – the midgut – is also a center of IIS. In particular, we have shown that ingested human insulin can activate IIS in midgut epithelial cells and significantly decrease the lifespan of *A. stephensi* mosquitoes [20], implying a direct relationship between exogenous insulin from the mammalian bloodmeal, activation of the midgut IIS, and lifespan. Therefore, we predicted that genetic manipulation of key IIS components in the midgut would offer a unique strategy for disrupting *P. falciparum* development while simultaneously decreasing the lifespan of the mosquito below the extrinsic incubation period (EIP) or the time required for malaria parasite development.

We genetically engineered *A. stephensi* to express an active variant of the mosquito Akt under the control of the midgut-specific carboxypeptidase (CP) promoter. As predicted, increased Akt signaling in the midgut significantly reduced malaria parasite development and mosquito lifespan. Both the number of infected mosquitoes and the average number of parasites per mosquito were reduced in transgenic mosquitoes relative to controls. In addition, transgenic mosquitoes had significantly shorter lifespans than non-transgenic siblings reared under identical conditions. These results demonstrate that manipulation of one signaling protein, Akt, in the mosquito midgut can affect both mosquito innate immunity and lifespan.

## Results

### Generation and characterization of the CP-myr-AsteAkt-HA transgenic mosquito line

**Figure 1:**
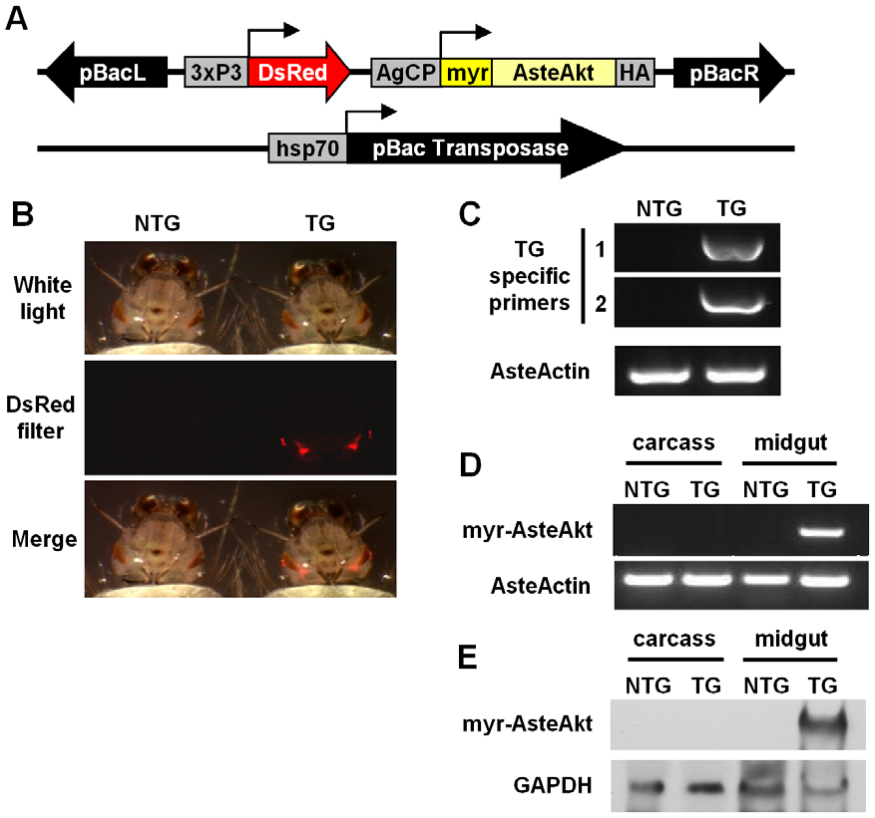
Generation of the *CP-myr-AsteAkt-HA* transgenic mosquito line and protein and transcript expression profile of the transgene in adult females. **A.** Schematic of the construct genetically engineered into *A. stephensi* mosquitoes. See text for a description of the construct. **B.** Comparison of transgenic (TG) and non-transgenic (NTG) siblings. Top panel: non-transgenic (left) and transgenic fourth instar larvae (right) under white light. Middle panel: non-transgenic and transgenic mosquitoes under fluorescence and a DsRed filter. Bottom panel: merge of top and middle panels. **C.** Two transgene-specific primer sets were used to amplify the transgene from the genomic DNA of transgenic and non-transgenic siblings. Primers to AsteActin were used to verify the integrity of the DNA. **D.** Total RNA was isolated from the midguts or carcasses (i.e., entire body minus midgut) of both transgenic (TG) and non-transgenic (NTG) mosquitoes and converted into cDNA. Transgene specific primers were used to amplify myr-AsteAkt. Primers to AsteActin were used to verify the integrity of the cDNA. **E.** Total protein was isolated from the midguts or carcasses of transgenic and non-transgenic mosquitoes, separated electrophoretically on a 12% SDS-PAGE gel. Proteins were blotted and then probed with anti-HA antibody or anti-GAPDH antibody to assess protein loading.

We generated a transgenic *A. stephensi* line overexpressing an activated form of Akt under the control of the midgut-specific CP promoter. Activated Akt was generated by a myristoylation sequence encoded at the amino terminus. An HA epitope at the carboxy terminus (myr-AsteAkt-HA) facilitated protein identification. The construct was inserted into the pBac[3XP3-DsRedafm] plasmid vector [21] for transformation into the *A. stephensi* genome (Fig. 1A). We injected approximately 4400 embryos with a mixture of the pBac[3XP3-DsRedafm]CP-myr-AsteAkt-HA donor plasmid and the phsp-pBac helper plasmid, resulting in approximately 176 adult mosquitoes whose progeny were screened for DsRed eye fluorescence (Fig. 1B). We isolated three F1 progeny with stable DsRed eye fluorescence, from which we established a stable line (Fig. 1C). Transgenic mosquitoes were maintained as a heterozygous line by outcrossing the mosquitoes in each generation to non-transgenic colony *A. stephensi*. A homozygous line was generated after approximately 20 generations of outcrossing and used to verify the viability of homozygous mosquitoes and to test the effect of increased Akt signaling on *P. falciparum* development in the midgut.

**Figure 2:**
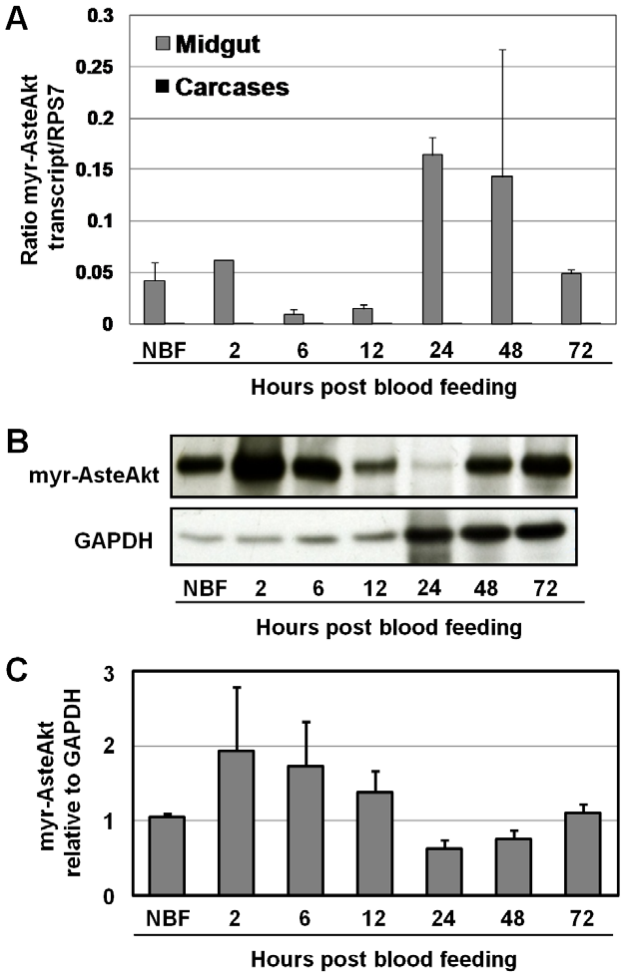
Expression profile of the transgene during a reproductive cycle. **A.** Total RNA was isolated from the midguts and carcasses of ten non-bloodfed (NBF) and ten bloodfed transgenic mosquitoes at 2 h, 24 h, 48 h and 72 h post-bloodfeeding and converted into cDNA. The 72 h sample was collected post-oviposition. Transgene specific qRT-PCR primers were used to amplify myr-AsteAkt from the cDNA; amplification of ribosomal protein S7 was used for normalization. The qRT-PCR experiments were performed in triplicate and replicated twice with separate cohorts of mosquitoes. Data are represented as means ± SEMs. **B.** Total protein was isolated from the midguts of transgenic mosquitoes at various time points during a reproductive cycle. Proteins were blotted and probed with anti-HA antibody or anti-GAPDH antibody to assess protein loading. **C.** Average expression of transgenic protein normalized to GAPDH loading controls and shown relative to levels in non-bloodfed mosquitoes (NBF). Data are represented as means ± SEMs from four replicates with separate cohorts of mosquitoes.

We assessed myr-AsteAkt-HA transcript expression levels during mosquito development and found that myr-AsteAkt-HA is primarily expressed in pupae and adult female stages (Figure S1, Text S1). In adult females, the myr-AsteAkt-HA transcript and protein were only detected within the midgut of transgenic mosquitoes (Figs. 1D and 1E). No expression was observed in the carcass of transgenic mosquitoes or in the midgut or carcass of non-transgenic mosquitoes. Surprisingly, we observed high levels of transcript (Fig. 2A) and protein (Fig. 2B) in the midguts of both non-bloodfed and bloodfed transgenic mosquitoes. Transcript expression increased slightly 2–6 h after bloodfeeding and increased dramatically between 24–48 h after the bloodmeal (Fig. 2A). Protein expression increased 2–12 h after the bloodmeal as would be expected for the CP promoter, but was reduced during the latter half of the reproductive cycle (24–48 h) (Figs. 2B and 2C).

### Membrane localization of the CP-myr-AsteAkt-HA protein

**Figure 3:**
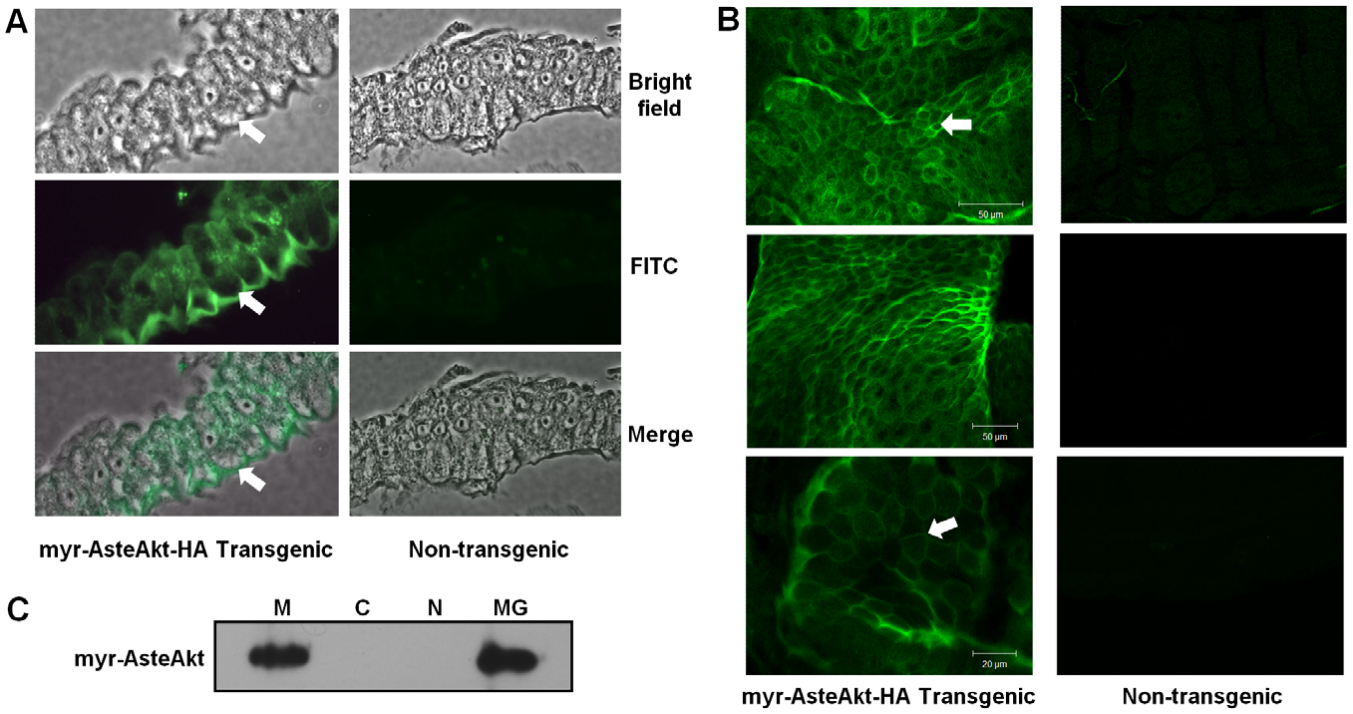
Membrane localization of the transgene. **A.** Immunocytochemistry of myr-AsteAkt-HA transgenic or non-transgenic paraffin embedded midgut sections using an anti-HA-fluorescein antibody. Images were acquired under brightfield illumination to visualize the midgut epithelial architecture or a FITC filter to visualize the overexpressed protein. To determine localization the images were merged. Arrows indicate the cell membrane of the midgut epithelial cells. Five midguts from transgenic and non-transgenic mosquitoes were analyzed. **B.** Immunocytochemistry of myr-AsteAkt-HA transgenic or non-transgenic midgut sheets using an anti-HA-fluorescein antibody. At least 10 midgut sheets from three separate cohorts of mosquitoes were analyzed by confocal microscopy. Three representative myr-AsteAkt-HA transgenic and non-transgenic midguts are shown. **C.** Membrane (M), cytoplasmic (C), and nuclear (N) fractions of transgenic mosquito midguts were prepared and these fractions, and an intact midgut sample (MG), were probed with anti-HA antibody. This immunoblot is representative of three experiments.

The myristoylation sequence at the amino terminus was expected to target myr-AsteAkt-HA to the cell membrane to be phosphorylated and activated by PDK1, eliminating the need for Akt binding to the upstream IIS component phosphoinositide (3,4,5)-trisphosphate and endogenous insulin signaling in general. To assess membrane localization of myr-AsteAkt-HA, we performed immunocytochemistry on both midgut sections (Fig. 3A) and whole midguts (Fig. 3B) of transgenic and non-transgenic mosquitoes using an anti-HA-fluorescein antibody. Strong staining of midgut epithelial cells was observed only in transgenic mosquitoes and no expression was observed in non-transgenic mosquitoes. A majority of the staining in midguts from transgenic mosquitoes was localized to the cell membrane as expected with the myristoylation sequence (Fig. 3A and 3B – white arrows). To confirm this result, we isolated the nuclei, cell membranes, and cytoplasm from midgut epithelia of transgenic mosquitoes and compared transgene protein levels in these fractions. The transgene protein was detected only in the cell membrane fraction at levels similar to those observed in an intact midgut (Fig. 3C).

### Activation of the IIS cascade in the mosquito midgut by human insulin and CP-myr-AsteAkt-HA

**Figure 4:**
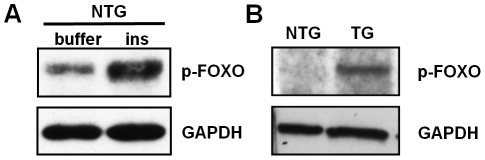
Increased FOXO phosphorylation due to dietary insulin and myr-AsteAkt-HA. **A.** Total protein from the midguts of non-transgenic (NTG) mosquitoes fed a bloodmeal either containing buffer or 1.7×10^−4^ µM human insulin were immunoblotted and probed with anti-phospho-FOXO1 (p-FOXO) antibody and anti-GAPDH to assess protein loading. **B.** A representative immunoblot of increased FOXO phosphorylation in myr-AsteAkt-HA transgenic mosquitoes compared to non-transgenic controls. Total protein from the midguts of transgenic (TG) and non-transgenic (NTG) mosquitoes maintained under identical conditions and fed only sucrose. The proteins were immunoblotted and probed with anti-phospho-FOXO1 (p-FOXO) antibody. The blot was then stripped and re-probed with and anti-GAPDH antibody to assess protein loading. This experiment was replicated three times.

FOXO1 is key transcription factor in the IIS cascade that is directly phosphorylated by Akt. Human insulin induced FOXO1 phosphorylation in the midguts of bloodfed, non-transgenic *A. stephensi* (Fig. 4A). In CP-myr-AsteAkt-HA-expressing mosquitoes, we also observed a marked increase in midgut FOXO1 phosphorylation relative to non-transgenic sibling mosquitoes even though a bloodmeal was not provided (Fig. 4B). This indicates that myr-AsteAkt-HA is active and capable of phosphorylating downstream IIS effectors. In sum, both human insulin and myr-AsteAkt-HA induced FOXO1 phosphorylation in vivo.

### The impact of myr-AsteAkt-HA expression on the prevalence and intensity of *P. falciparum* infection in the mosquito

**Figure 5:**
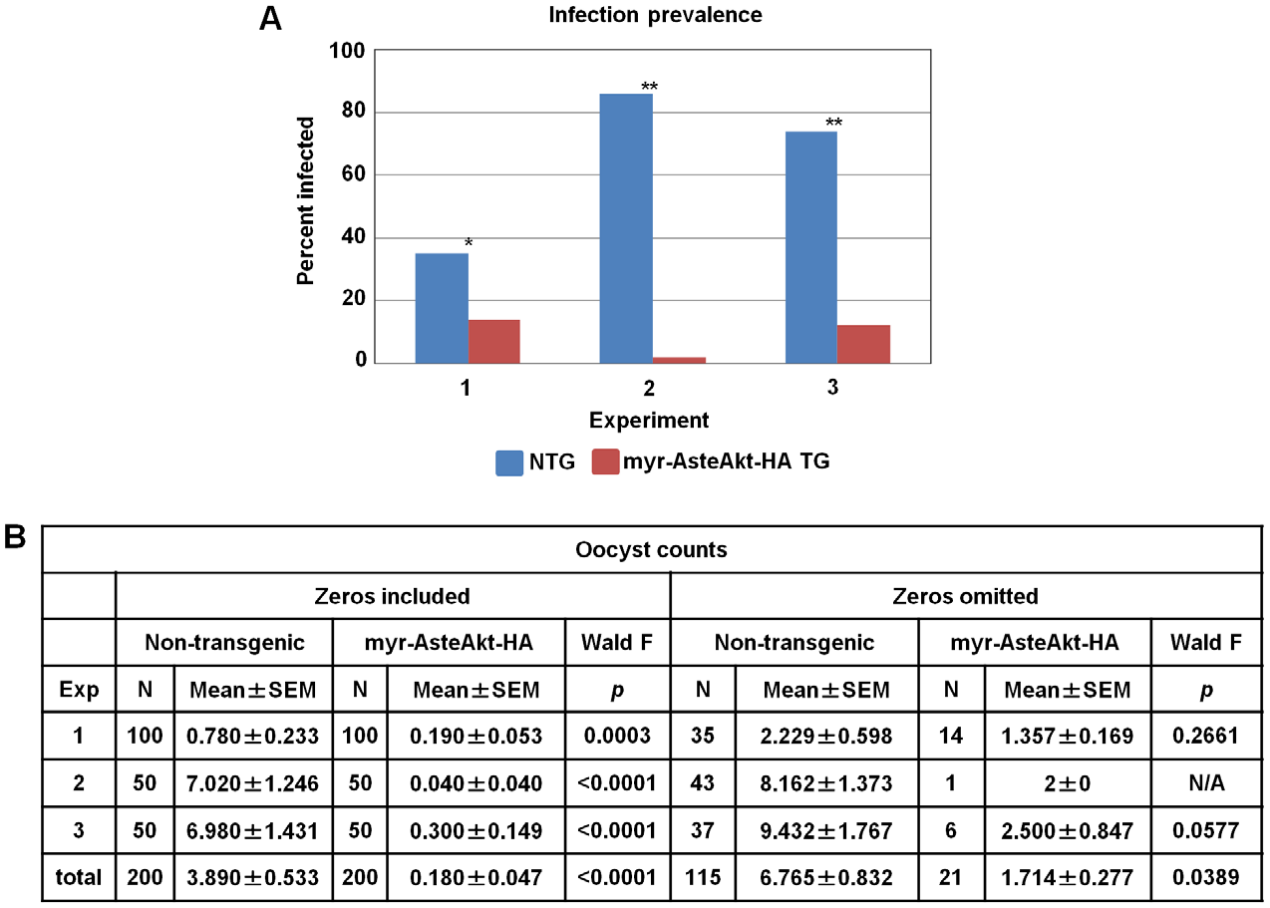
Resistance of heterozygous transgenic and non-transgenic mosquitoes to *P. falciparum* infection. Heterozygous transgenic (TG) and non-transgenic (NTG) sibling mosquitoes were provided with an artificial bloodmeal containing *P. falciparum* NF54 gametocytes. Ten days after infection, the midguts were dissected and the number of *P. falciparum* oocysts counted. The experiment was replicated three times with separate cohorts of mosquitoes. **A.** Infection prevalence was defined as the percentage of mosquitoes that had at least one oocyst on the midgut. Parasite prevalence was significantly lower in transgenic mosquitoes compared to the nontransgenic control (** indicates p<0.0001, * indicates p = 0.0008). **B.** Summary statistics of the parasite data from all three experiments including zeros or excluding zeros (mosquitoes without oocysts). Analyses were performed for each replicate separately and for all three replicates combined. When zeros were omitted, the small number of transgenic mosquitoes infected with at least one parasite (n = 1–14) obviated statistical analysis.

Increased Akt activity in the midgut epithelium led to major reductions in both the percentage of mosquitoes infected with *P. falciparum* and the number of oocysts in the midguts of infected mosquitoes (Fig. 5). The percentage of mosquitoes with one or more oocysts decreased from an average of 58.5% (36–86%) in non-transgenic controls to 10.5% (2–14%) in heterozyogous myr-AsteAkt mosquitoes (Fig. 5A, p<0.0001; pooled across replicates). Similarly, the intensity of infection was reduced by 95.6% from an average of 3.9 oocysts/midgut (0–45; n = 200) in non-transgenic controls to 0.18 (0–6; n = 200) in myr-AsteAkt mosquitoes (Fig. 5B). This rate of inhibition is higher than rates reported for other anti-parasite effector molecules, including SM1 (81.6%), PLA2 (87%), anti-HAP2 (81.1%), and anti-chitinase (91.3%) [22]–[25].

**Figure 6:**
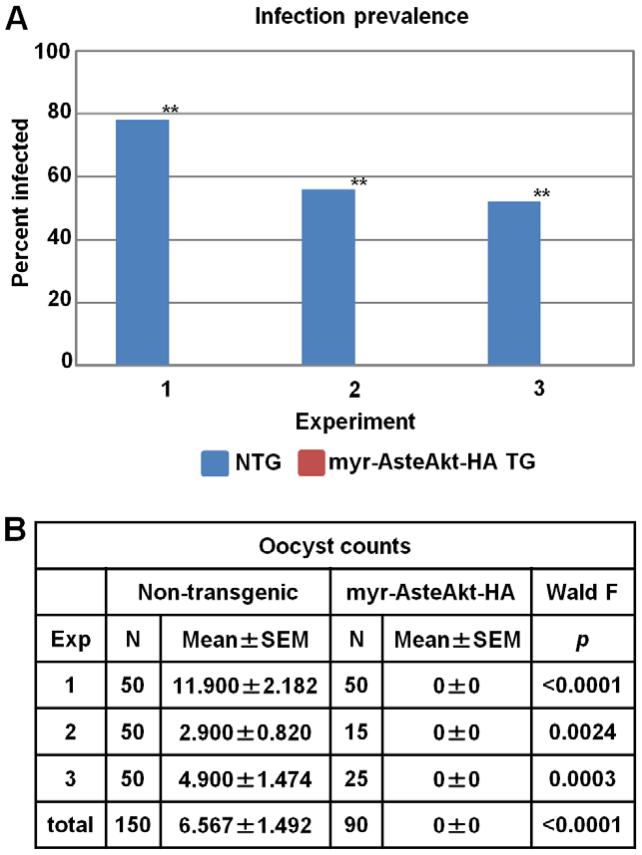
Resistance of homozygous transgenic and non-transgenic mosquitoes to *P. falciparum* infection. Homozygous transgenic (TG) mosquitoes and non-transgenic (NTG) mosquitoes were provided with an artificial bloodmeal containing *P. falciparum* NF54 gametocytes. Ten days after infection, the midguts were dissected and the numbers of *P. falciparum* oocysts were counted. **A.** Infection prevalence was defined as the percentage of mosquitoes that had at least one oocyst on the midgut. Homozygous transgenic mosquitoes were significantly less prone to infection (** indicates p<0.0001). **B.** Summary statistics of parasite data from all three experiments. Analyses were performed for each replicate separately and for the three replicates combined.

We also assessed the effect of doubling myr-AsteAkt expression by establishing a homozygous transgenic line. *P. falciparum* infection in the homozygous line was completely blocked, with no viable oocysts observed in any of the midguts (Fig. 6; n = 90). In contrast, 62% of control mosquitoes had at least one oocyst, with an average of 6.6 parasites per midgut (0–76; n = 150). A recent study demonstrated that the combination of two effector molecules, defensin A and cercropin A, was capable of completely blocking the development of the avian malaria parasite *Plasmodium gallinaceum* in *Aedes aegypti* [26]. However, our data constitute the first example of a single effector molecule in a transgenic mosquito completely blocking invasion by the human malaria parasite.

### The impact of myr-AsteAkt-HA expression on mosquito lifespan

**Figure 7:**
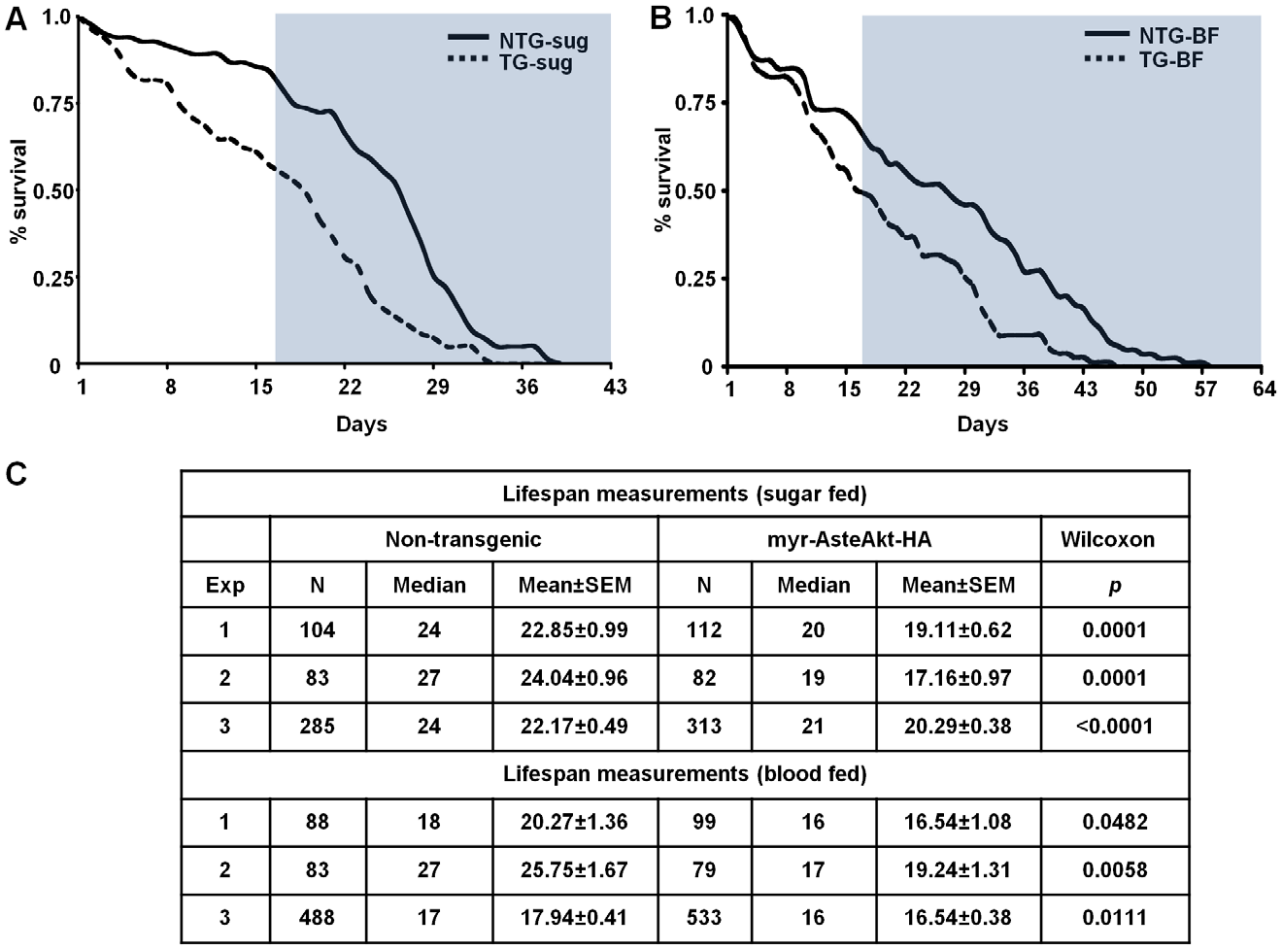
Lifespan experiments of sugarfed or bloodfed transgenic and non-transgenic mosquitoes. **A.** A representative survivorship curve comparing transgenic (TG) and non-transgenic (NTG) siblings reared under identical conditions and provided with only a 10% sucrose solution. Lifespan experiments were replicated three times with separate cohorts of mosquitoes. **B.** A representative survivorship curve comparing transgenic (TG) and non-transgenic (NTG) siblings reared under identical conditions and provided with weekly bloodmeals and a 10% sucrose solution. Lifespan experiments were replicated three times with separate cohorts of mosquitoes. **C.** Summary of the samples sizes, medians, means, and statistical significance for sugarfed and bloodfed mosquitoes.

We hypothesized that increased activation of IIS due to expression of myr-AsteAkt-HA in the midgut would alter the lifespan of sugarfed and bloodfed mosquitoes relative to non-transgenic controls. In contrast to bloodfeeding, mosquitoes provided with sugar only do not enter a reproductive cycle or produce eggs. In replicated assays, sugarfed transgenic mosquitoes lived an average of 18.85 (17.16–20.29) days compared to 23.02 (22.17–24.04) days for non-transgenic control siblings, a decrease of nearly 20% (Fig. 7A; p<0.0001 to p = 0.0001). This same trend was observed in transgenic mosquitoes provided with weekly bloodmeals and given the opportunity to produce a weekly clutch of eggs. Bloodfed transgenic mosquitoes survived an average of 17.44 (16.54–19.24) days compared with 21.32 (17.94–25.75) days for non-transgenic control siblings, a reduction of more than 18% (Fig. 7B; p = 0.0058 to 0.0482).

An important measure for malaria control is the percent of the population that survives long enough to transmit the malaria parasite. If one assumes that (1) a female mosquito finds a mate on the first day after adult eclosion, (2) she acquires an infective bloodmeal on the second day, and (3) the parasite develops and invades the salivary glands 14 days after taking the infected bloodmeal, then that mosquito must survive a minimum of 16 days to successfully transmit *P. falciparum* (blue areas Figs. 7A and 7B). Under our conditions, an average of 59% of the non-transgenic mosquitoes given weekly bloodmeals were still alive at day 16 compared to 44% of the myr-AsteAkt transgenic mosquitoes. Comparing the area under the lifespan curves of the transgenic and non-transgenic siblings after 16 days, we observed a 53% reduction in sugarfed mosquitoes and a 48% reduction in bloodfed mosquitoes. This indicates that the population of competent malaria vectors can be reduced by half with a modest 20% reduction in lifespan.

Fitness costs due to the generation of the transgenic line or the transgene itself were likely minimal due to transgene insertion into non-coding sequence (Figure S2, Text S1) and repeated out-crossing to non-transgenic mosquitoes. In addition, lifespan studies were performed using sibling transgenic and non-transgenic mosquitoes to minimize genetic differences and were performed with sibling mosquitoes reared as larvae in the same pans of water and separated as pupae to minimize environmental differences.

### The impact of myr-AsteAkt-HA expression on mosquito reproduction

Insulin signaling regulates reproduction in a wide range of organisms. In insects, including mosquitoes, IIS has been shown to regulate steroidogenesis in the ovaries and vitellogenesis in the fat body [27], [28]. Although IIS in the midgut has not previously been implicated in the regulation of reproduction, we examined whether any differences in egg production between the transgenic and non-transgenic siblings could be detected. For the five replicates in which zero egg counts were recorded, there were no significant differences between transgenic and non-transgenic females in the number of eggs laid (Table 1). Among those that laid eggs, only one replicate of the six indicated a significant difference between non-transgenic and transgenic females. The remaining replicates indicated no difference between genotypes (Table 1). There was no difference between genotypes in whether or not females laid eggs.

**Table 1:** Egg counts for transgenic and non-transgenic females.

Exp	Zeros included					Zeros omitted				
	Non-transgenic		myr-AsteAkt-HA		Wilcoxon	Non-transgenic		myr-AsteAkt-HA		Wilcoxon
	N	Mean±SEM	N	Mean±SEM	*p*	N	Mean±SEM	N	Mean±SEM	*p*
1						10	83.20±11.45	15	78.67±5.19	0.405
2	18	35.83±11.16	25	28.83±7.87	0.699	7	92.14±6.53	10	69.20±8.35	0.057
3	28	18.14±5.70	28	10.57±3.39	0.442	9	56.44±8.46	8	37.00±3.90	0.021
4	19	10.63±6.32	20	19.50±7.27	0.341	3	67.33±19.15	6	65.00±8.79	0.606
5	28	26.11±7.99	28	23.36±6.98	0.812	9	81.22±10.49	9	72.67±7.98	0.289
6	36	70.36±9.75	36	66.92±7.95	0.618	23	110.13±6.13	26	92.63±5.22	0.051

Myr-AsteAkt-HA transgenic and non-transgenic siblings were allowed to bloodfeed. Fully engorged females were placed into individual cages, provided with an oviposition substrate, and the number of eggs laid counted. Results are presented for each replicate separately, including the number of females tested (N), the mean number of eggs laid ± SEMs, and the *p*-value of the Wilcoxon test. Egg count data were subjected to a nonparametric Wilcoxon test. The numbers of females that did not lay eggs in the first replicate were not recorded.

To ensure that differences in egg production were not due to the size of the blood meal, the amount of blood ingested was also compared between transgenic and non-transgenic females. For all five replicates, there was no significant difference between genotypes in the amount of blood ingested (Figure S3A and S3B, Text S1). In addition, no obvious differences were observed between transgenic and non-transgenic sibling mosquitoes in the amount of undigested BSA remaining at 24 h after blood ingestion (Figure S3C, Text S1).

## Discussion

Mosquitoes require a bloodmeal to initiate a reproductive cycle and produce eggs. Within this bloodmeal are insulin, insulin-like growth factor 1, and various other factors that circulate in the blood of the human host. Our previous work demonstrated that some of these factors, including human insulin and human TGF-β1, activate mitogen-activated protein (MAP) kinase and phosphoinositide-3 kinase (PI3K) signaling cascades in the mosquito midgut [20], [29]. Here, we used transgenesis to overexpress a key component of the IIS cascade, Akt, in the *A. stephensi* midgut to induce signaling independent of exogenous insulin. We observed significant reductions in both the prevalence and intensity of *P. falciparum* infections in transgenic mosquitoes following the consumption of an infective bloodmeal. We also observed a reduction in lifespan consistent with that observed in insulin-fed *A. stephensi* [20], indicating that the mosquito midgut plays a central role in regulating lifespan.

Myristoylated Akt localized to the midgut epithelial cell membrane in transgenic *A. stephensi* (Fig. 3) where it was activated to subsequently phosphorylate the downstream effector protein FOXO1 (Fig. 4C). This parallels FOXO1 phosphorylation in the midguts of mosquitoes fed bloodmeals containing insulin (Fig 4A). Taken together, these results suggest that the mechanisms of parasite and lifespan reduction observed in CP-myr-AsteAkt-HA transgenic mosquitoes are dependent on the activation of the PI3K/Akt/FOXO arm of the IIS cascade. It is noteworthy that Akt has been defined as “a critical signaling node within all cells of higher eukaryotes and one of the most important and versatile protein kinases at the core of human physiology and disease [30].” Akt has more than 100 experimentally verified substrates and broad crosstalk between a variety of biologically important signal transduction pathways. Thus, the mechanisms through which tissue-specific Akt overexpression regulates innate immunity and lifespan are likely to be complex [30].

A carboxypeptidase promoter drives the myr-AsteAkt-HA transgene, so we expected expression to rise shortly after a bloodmeal was consumed and to be midgut-specific. Expression of myr-AsteAkt-HA was indeed specific to the midgut (Fig. 1D and E), but the timing of expression was unexpected since both transcript and protein were observed even in the absence of a bloodmeal (Fig. 2). As expected for a gene regulated by a carboxypeptidase promoter, however, protein expression increased following ingestion of the bloodmeal. Leaky transgene expression has been observed with this promoter, resulting in expression prior to bloodfeeding [31] or late in the reproductive cycle [32]. The process of generating a transgenic mosquito strain could also explain the unexpected expression patterns. For example, the transgene may have inserted near an enhancer DNA sequence, resulting in greater gene and protein expression [33]. Although this pattern of myr-AsteAkt-HA expression was unexpected, it was ultimately advantageous because increased insulin signaling is maintained for the apparent duration of adult female life and does not depend on consumption of a bloodmeal for activity. Thus, the anti-parasite activity and lifespan effects of myr-AsteAkt-HA will occur regardless of the timing and quantity of bloodmeals that are consumed by a transgenic mosquito.

Increased insulin signaling in the mosquito midgut, whether through ingestion of exogenous insulin [20] or overexpression of active IIS proteins such as Akt, can significantly reduce mosquito lifespan and inhibit *P. falciparum* development. Importantly, we observed that increased AsteAkt expression in the homozygous line increased parasite resistance to the point that oocyst formation on the midgut was completely blocked. Although it will likely be necessary to deploy heterozygous mosquitoes for any future transmission blocking strategy, our data suggest that an increase in myr-AsteAkt expression, possibly through manipulation of the promoter or transgene insertion site, could yield heterozygous mosquitoes that are resistant to *P. falcipaurm* infection. Lifespan reduction can also impact malaria parasite prevalence based on the combined effects of a relatively short natural lifespan of *A. stephensi* [4]–[6] and a relatively lengthy parasite development time. In particular, models of vector competence routinely demonstrate that the daily probability of survival is the single most important factor in determining how effectively a mosquito transmits a pathogen [34]. All else being equal, even modest reductions in lifespan will have significant effects on disease transmission.

In summary, we have developed a novel mechanism to reduce the transmission of the human malaria parasite *P. falciparum*. This approach is based on the manipulation of two key physiological parameters, lifespan and innate immunity, through activation of a single signaling protein, Akt. Increased Akt activity significantly reduced infection prevalence in the mosquito host at the same time that it reduced the infective period of the mosquito lifespan. A multi-component approach to transgenesis focused on manipulation of the IIS cascade could be combined with overexpression of additional anti-parasite effectors to effectively block parasite transmission, reduce lifespan, and increase fecundity. Perhaps more importantly, a multi-component approach could prevent the escape of adaptive parasite variants, providing a powerful new tool for malaria control.

## Materials and Methods

### Mosquitoes

*Anopheles stephensi* mosquitoes were maintained at 28°C, 75% RH, on a 16∶8 light∶dark photoperiod. Larval mosquitoes were fed cat food pellets (Purina). Adult mosquitoes were fed ad libitum on a 10% dextrose solution. Porcine blood (UA Meat Science facility) supplemented with sodium citrate (0.38%) and warmed to 37°C was used for colony maintenance and bloodfeeding experiments. For feeding experiments, engorged females were separated from unfed and partially fed mosquitoes and maintained on 10% dextrose until needed. Females used for post-oviposition studies were transferred 48 h post-blood meal to a new container and allowed to oviposit on moistened filter papers overnight.

### Generating the CP-Myr-AsteAkt-HA transgenic line

The *A. gambiae* carboxypeptidase (CP) promoter was kindly provided by Dr. Luciano Moreira [32]. The 5′ promoter was amplified with primers designed to remove the signal peptide, start methionine and Kozak consensus sequence, and to add XhoI and NotI restriction digest sites. The modified 5′ CP promoter was ligated into the phsp-pBac shuttle plasmid using XhoI and NotI sites [21]. The SV40 3′ UTR was ligated into the EcoRI site of phsp-pBac. A Kozak consensus sequence (CCAACCATGG) and Src myristoylation sequence (MGSSKSKPKDPSQR) were added to the 5′ end of AsteAkt, while an HA epitope (YPYDVPDYA) was added to the 3′ end. This construct was inserted it into the phsp-pBac shuttle containing the CP promoter and SV40 3′ UTR. Finally, the CP-myr-AsteAkt-HA-SV40 construct was ligated into the pBac[3XP3-DsRedafm] construct to generate the pBac[3XP3-DsRedafm]CP-myr-AsteAkt-HA plasmid for injection into *A. stephensi* embryos.

Donor (500 ng/µl) and helper (200 ng/µl) plasmids were injected into newly oviposited embryos, which were then reared to adulthood and screened for transgene insertion as described by Lobo *et al* [35]. Lifespan and reproduction experiments were initiated only after five generations of outcrossing the transgenic line to a non-transgenic lab strain. Crosses between heterozygous transgenic and non-transgenic mosquitoes produced a 50/50 ratio of transgenic to non-transgenic siblings.

### Expression analysis of the myr-AsteAkt-HA transcript and protein

Midguts and carcasses (whole body without midgut) were collected from ten transgenic females prior to bloodfeeding and at 2, 6, 12, 24, 48, and 72 h PBM. Total RNA was extracted using RNeasy kit (Qiagen), treated with DNase 1 (Fermentas) and cDNA was synthesized using High Capacity cDNA ReverseTranscription Kit (Applied Biosystems) with random hexamer primers. Quantitative real-time PCR (qRT-PCR) was performed using Maxima SYBP Green/ROX qPCR master mix (Fermentas) and an ABI 7300 real-time PCR system. Myr-AsteAKT-HA-specific primers (forward: 5′-TTACCGGTGAAAGTGTGGAGCTGA-3′; reverse: 5′-AAGCGTAATCTGGCACATCGTATGG-3′; efficiency - 98%) were used to detect myr-AsteAKT-HA in midguts and carcasses. Myr-AsteAKT-HA expression was normalized to ribosomal protein S7 expression. qRT-PCR reactions were performed in triplicate and the experiment was replicated twice with separate cohorts of mosquitoes.

Immunoblots were performed with one midgut equivalent of protein as previously described [36]. Myr-AsteAkt-HA protein levels were detected using an anti-HA antibody (1∶20,000 dilution; Roche). RT-PCR and immunoblot assays were replicated three times with separate cohorts of mosquitoes.

### Activation of the IIS cascade in *A. stephensi* midguts by human insulin and myr-AsteAkt-HA

A total of 100 3- to 5-day-old female *A. stephensi* mosquitoes were fed artificial bloodmeals supplemented with 1.7×10^−3^ µmol of human insulin or an equivalent volume of insulin buffer as described in Kang *et al* [20]. Immunoblot analyses of protein phosphorylation from 60 midguts per treatment group were conducted as previously described [20]. Midgut samples were probed with anti-phospho FOXO1A/FOXO3A antibody (1∶1000 dilution, Millipore) or an anti-GADPH antibody (1∶10,000 dilution, Abcam) to assess protein loading.

In the CP-myr-AsteAkt transgenic mosquitoes midguts were subjected to immunoblot analysis as described above, and were probed with anti-phospho-FOXO1A antibody (1∶10,000 dilution; Millipore). Five midgut equivalents of protein were used per lane. Blots were stripped and re-probed with an anti-GADPH antibody (1∶40,000 dilution, CST) to assess protein loading.

### Membrane localization of myr-AsteAkt-HA

For whole mount immunocytochemistry studies, midguts were dissected from 10 transgenic and 10 non-transgenic mosquitoes in 1× *Aedes* saline (125 mM NaCl, 5mM KCl, 1.85 mM CaCl_2_, pH 6.5) and opened into a midgut sheet. Immunocytochemistry was performed as described by Riehle and Brown [37], except that an anti-HA antibody conjugated to fluorescein (1∶1000, Roche) was used without a secondary antibody. All samples were imaged at identical settings to facilitate comparison. Experiments were replicated a minimum of three times with separate cohorts of mosquitoes. For immunocytochemistry using paraffin embedded sections, midguts were dissected from 10 transgenic and 10 non-transgenic mosquitoes in 1× *Aedes* saline and 10× Complete protease inhibitors (Roche). Midguts were immediately transferred to 4% paraformaldehyde in PBS for 2 h at RT and then stored in 70% EtOH at 4°C until embedded. The midguts were embedded in paraffin at the University of Arizona histology center and cut to obtain 4.5–5 µM sections. The paraffin was removed by two xylene washes of 10 min and the samples were hydrated through a series of solutions of decreasing ethanol concentration (100, 95, 70, 50 and 30%). The tissues were washed in PBS with 0.1% Tween 20 (PBS-T) and then blocked in a solution of 2% BSA/PBS-T for 2 h at RT. The slides were incubated overnight in a humid chamber with a 1∶500 dilution of the anti-HA antibody conjugated to fluorescein. The tissues were washed 3× in PBS-T for 15 min at RT and observed under a Nikon Eclipse E600 fluorescent microscope. Images were acquired using a SPOT camera system (Diagnostic Instruments Inc) at identical settings for all fluorescent images.

To verify the subcellular localization of myr-AsteAkt-HA, we prepared midgut cell membranes, nuclei and cytoplasm from midguts from transgenic and non-transgenic *A. stephensi* as described by Brown *et al* [38]. The three sub-cellular fractions were subjected to immunoblot analysis using the anti-HA antibody as described above with replicated samples from three separate cohorts of mosquitoes.

### *P. falciparum* studies

Cultures of *P. falciparum* NF54 were initiated at 1% parasitemia in 10% heat-inactivated human serum, and 6% washed human RBCs in RPMI 1640 with HEPES (Gibco) and hypoxanthine. Stage V gametocytes were evident by day 15 and exflagellation was evaluated on the day prior to and the day of mosquito feeding. For our assays, 5-day old female transgenic and non-transgenic *A. stephensi* were fed on mature gametocyte culture diluted with human erythrocytes and heat-inactivated serum. On day 10, midguts from fully gravid females were dissected in PBS and stained with 1% mercurochrome/PBS to visualize *P. falciparum* oocysts. Oocysts were counted for each midgut and mean oocysts per midgut (infection intensity) and percentages of infected mosquitoes (infection prevalence; infection = at least one oocyst) were calculated from all dissected mosquitoes.

### Lifespan studies

Transgenic mosquitoes heterozygous for the CP-myr-AsteAkt-HA construct were mated with non-transgenic mosquitoes to generate 50% transgenic and 50% non-transgenic mosquitoes. The resulting larvae were reared together under identical conditions and separated based on DsRed fluorescence in the eyes of pupae under a fluorescent stereomicroscope. Female mosquitoes were separated into four treatment groups: transgenic bloodfed, transgenic sugarfed, non-transgenic bloodfed, and non-transgenic sugarfed. Bloodfed mosquitoes were given weekly bloodmeals throughout their entire adult life in addition to 10% dextrose ad libitum, while sugarfed mosquitoes were only provided 10% dextrose ad libitum. Daily mortality for each treatment was recorded and dead mosquitoes were removed until all mosquitoes had perished. These experiments were replicated twice. A third experiment was conducted using approximately 500 mosquitoes per treatment to verify the initial results.

### Reproduction studies

Transgenic CP-myr-AsteAkt-HA females and their non-transgenic siblings were mated with colony males shortly after emergence. At 5–7 days post-emergence, females were starved overnight and then fed a blood meal. Fully engorged females were placed into individual cages and provided with an oviposition site and 10% dextrose ad libitum. Oviposition sites were removed 72 h after bloodfeeding and the numbers of eggs were counted. The experiment was repeated six times with separate cohorts of mosquitoes. In the first experiment, data were recorded only for those mosquitoes that laid eggs. In subsequent replicates, the number of individuals that did not lay eggs was recorded. For each replicate, the non-normally distributed egg counts were first analyzed using a Wilcoxon test to determine if there was a significant difference between transgenic and non-transgenic females.

### Statistical analyses

Parasite prevalence and oocyst numbers were analyzed to determine whether transgenic mosquitoes were more resistant than their nontransgenic siblings. The data were analyzed in two ways, first by determining whether genotype was an important predictor of resistance within replicates and also pooled across replicates. This allowed us to infer, in part, why replicates within the same experiment differed. In contrast, for the pooled data sets, we included replicate as a random effect to control for inter-replicate variation without explicitly estimating their mean values.

Parasite prevalence data were analyzed to determine whether infection status (infected or not) depended on genotype. The data were analyzed for each replicate separately using a logistic regression with genotype as a fixed effect. Data for all replicates were then combined and analyzed using a generalized linear mixed model with replicate and genotype included as a random and fixed effect, respectively, in the model. Significant differences were detected using a Wald χ^2^ statistic.

Oocyst counts were square-root transformed to correct for overdispersion prior to using a generalized linear mixed model analysis. Data were first analyzed for each replicate separately to test for the fixed effect of genotype. The data were then combined across replicates and analyzed using replicate as a random effect and genotype as a fixed effect. Significant differences were detected using Wald's *F* statistic.

Analysis of survival curves was conducted using the Kaplan Meier method [39] and significant differences were detected using the Wilcoxon test as previously described [20].

## Supporting Information

Text S1 Supporting information text.
(0.03 MB DOC)

Figure S1 Transcript expression profile of the transgene during mosquito development. Transcript expression at various developmental stages of transgenic mosquitoes (2^nd^ instar larvae, early and late 4^th^ instar larvae, newly eclosed pupae and late (24 h) pupae, and adult males and females). The experiment was replicated four times with separate cohorts of mosquitoes.
(0.09 MB TIF)

Figure S2 Gene sequence of inverse PCR fragment. **A.** A schematic of the inverse PCR product sequence. Transgenic genomic DNA was cut with *Mbo*I and was self-ligated to form circularized DNA which was used as a template for PCR with pBac-specific primers. As expected, the amplified product (97 bp) from the putative insertion site was flanked with known pBac sequence. **B.** Putative insertion site sequence and translation in all 6 frames. Translation is presented using the one-letter symbol for each amino acid. Stop codons are represented using a dash (-).
(0.16 MB TIF)

Figure S3 Bloodmeal ingestion and digestion are not affected in myr-AsteAKT-HA transgenics. **A and B.** Transgenic myr-AsteAKT-HA females ingested the same amount of blood as non-transgenic siblings. Average blood intake was calculated as a difference between an average weight of engorged females in the given weight group before and after bloodfeeding. The p-values reflect difference in the weight between groups of non-transgenic and transgenic mosquitoes within one weight category after a bloodmeal. **C.** Immunoblot analysis of midguts from five individual myr-AsteAKT-HA transgenic and non-transgenic females 24 h after feeding on bovine blood did not detect obvious differences in the amount of full length BSA remaining in the gut. Each lane was loaded with 0.1 midgut equivalent and probed with an anti-BSA antibody.
(0.19 MB TIF)
