# Correction: Activation of *Akt* Signaling Reduces the Prevalence and Intensity of Malaria Parasite Infection and Lifespan in *Anopheles stephensi* Mosquitoes

**DOI:** 10.1371/annotation/738ac91f-8c41-4bf5-9a39-bddf0b777a89

**Published:** 2010-08-10

**Authors:** Vanessa Corby-Harris, Anna Drexler, Laurel Watkins de Jong, Yevgeniya Antonova, Nazzy Pakpour, Rolf Ziegler, Frank Ramberg, Edwin E. Lewis, Jessica M. Brown, Shirley Luckhart, Michael A. Riehle

Information was missing from the Funding section. The correct funding information is as follows: This project was supported by grants from the National Institute of Allergy and Infectious Diseases (RO1A1073745 and RO1AI080799). The funders had no role in study design, data collection and analysis, decision to publish, or preparation of the manuscript.

