# Supplementary material for: Activation of Akt Signaling Reduces the Prevalence and Intensity of Malaria Parasite Infection and Lifespan in Anopheles stephensi Mosquitoes
Source: PLoS Pathog. 2010 Jul 15;6(7):e1001003. doi: 10.1371/journal.ppat.1001003 (PMC2904800; doi:10.1371/journal.ppat.1001003)
Supplement: Text S1 — Supporting information text. (0.03 MB DOC) [file ppat.1001003.s001.doc]

**Supporting Information**

*Characterization of the transgene across developmental stages*

Transcript expression of the transgene was examined across mosquito developmental stages. Total RNA was isolated from various developmental stages of transgenic mosquitoes (2^nd^ instar larvae, early and late 4^th^ instar larvae, newly eclosed pupae and late (24 h) pupae, and adult males and females) and converted into cDNA. Transgene-specific primers (described in Materials and Methods) were used to amplify CP-myr-AsteAkt-HA. AsteActin was used as a control. The transgene transcript was expressed predominantly during the pupal and adult stages, with minimal transcript expression during late larval development (Fig. S1). Transcript was not detected in earlier larval stages**.**

*Identification of the genomic insertion site of CP-myr-AsteAkt-HA*

The insertion site of the CP-myr-AsteAkt-HA transgene was determined using inverse PCR. Briefly, genomic DNA from transgenic mosquitoes was digested to completion with *Mbo*I. Fragments were self-circularized and PCR amplified using primers 5’-TATCGGTCTGTATATCGAGGTT-3’ (forward) and 5’-CGCATGATTATCTTTAACGTACGTCAC-3’ (reverse) under the following conditions: 95º C 2 min; 95º C 30 sec - 50º C 60 sec - 72º C 2 min: 35 cycles; 72º C 10 min. After resolution through 1.5% agarose, amplified fragments were excised and submitted for direct sequencing at the Arizona Research Laboratories Division of Biotechnology Genomic Analysis and Technology Core Facility (<http://gatc.arl.arizona.edu/>).

Two lines of evidence suggest that the CP-myr-AsteAkt-HA transgene was inserted into sequence with features of a noncoding region of the *A. stephensi* genome. The sequence flanking the unknown sequence was 100% identical to multiple pBac cloning vectors, confirming that the unknown sequence was flanked by known pBac sequence. The 98 bp unknown region was rich in stop codons in all three translated frames and did not have similarity to any insect gene in the NCBI database or the *A. gambiae* genome. We suspect that the region of the *A. stephensi* genome that the transgene inserted into arose independently since the split between *A. stephensi* and *A. gambiae*.

To more rigorously determine the position of the insertion site, we searched for significant regions of similarity between the 97 bp sequence and *A. stephensi* 454-generated genome sequences kindly provided by Zhijian Tu (unpublished data). The insertion site sequence matched exactly with one contig (containing the 97 bp flanking sequence and the remainder of the 454 generated fragment). No significant matches to this contig were found in GenBank. A search for the translated contig in the NCBI translated protein database yielded a match to one protein, the transcript of *Drosophila ananassae* gene GF18460 (accession number XP_001954819). This matching region had a high e-value (0.28), shared low sequence similarity (32%), and was approximately 1.4 kb away from the transgene insertion. Taken together, these results suggest that the transgene inserted into a noncoding region of the *An. stephensi* genome.

*Bloodmeal size and digestion in myr-AsteAkt-HA transgenic and non-transgenic siblings*

Myr-AsteAKT-HA transgenic females (3-5 d) and non-transgenic siblings were starved overnight and weighed. Females were provided access to 10% dextrose for ~12 h, subsequently starved again overnight, and then provided a bloodmeal. Mosquitoes that fed to repletion were immediately weighed and compared with their prefed weight to determine the amount of blood consumed. No significant differences were observed between myr-AsteAKT-HA transgenic females and non-transgenic siblings indicating that the transgene did not adversely affect blood intake (Fig S3A and S3B).

To assess digestion of the bloodmeal myr-AsteAKT-HA transgenic and non-transgenic females weighing between 1.4-1.5 mg were provided bovine blood. Individual midguts from fully engorged females were dissected in TBS buffer containing 10x Complete Protease Inhibitor (Roche) 24 h after blood feeding. Midguts were homogenized in lysis buffer (PBS, 1.0% Triton X-100, 2% SDS, 10X Complete Protease Inhibitor) and 0.1 midgut equivalents were loaded onto a 10% Precise SDS-PAGE gel (Thermo Scientific). Immmunodetection of BSA was performed as described by Isoe et al. 2009. Briefly, fractionated proteins were transferred to a nitrocellulose membrane (LI-COR Biosciences) and probed with an anti-BSA polyclonal antibody (1:2000; Gallus Immunotech). The secondary antibody used was IRDye 680 donkey anti-chicken secondary antibody (1:10,000; LI-COR). The proteins were detected with an Odyssey infrared imaging system (LI-COR). No observable differences in the amount of intact BSA remaining after 24 h of digestion indicated that the transgene did not affect bloodmeal digestion.
