# Supplementary figures and images for: Activation of Akt Signaling Reduces the Prevalence and Intensity of Malaria Parasite Infection and Lifespan in Anopheles stephensi Mosquitoes
Source: PLoS Pathog. 2010 Jul 15;6(7):e1001003. doi: 10.1371/journal.ppat.1001003 (PMC2904800; doi:10.1371/journal.ppat.1001003)

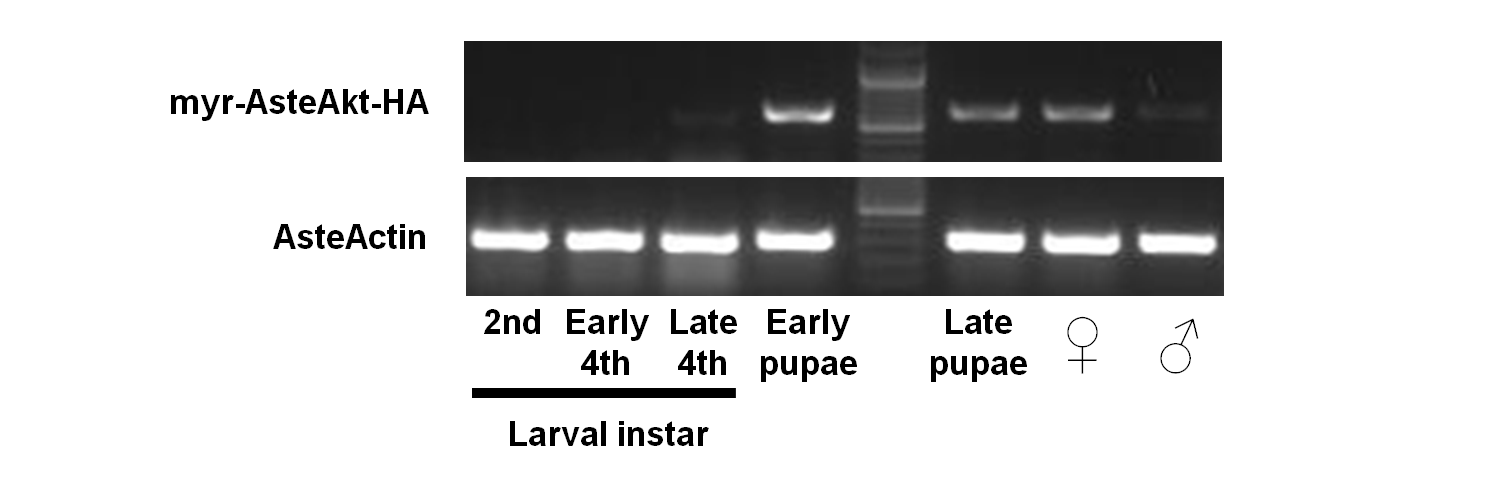

Supplement: Figure S1 — Transcript expression profile of the transgene during mosquito development. Transcript expression at various developmental stages of transgenic mosquitoes (2nd instar larvae, early and late 4th instar larvae, newly eclosed pupae and late (24 h) pupae, and adult males and females). The experiment was replicated four times with separate cohorts of mosquitoes. (0.09 MB TIF) [file ppat.1001003.s002.tif]

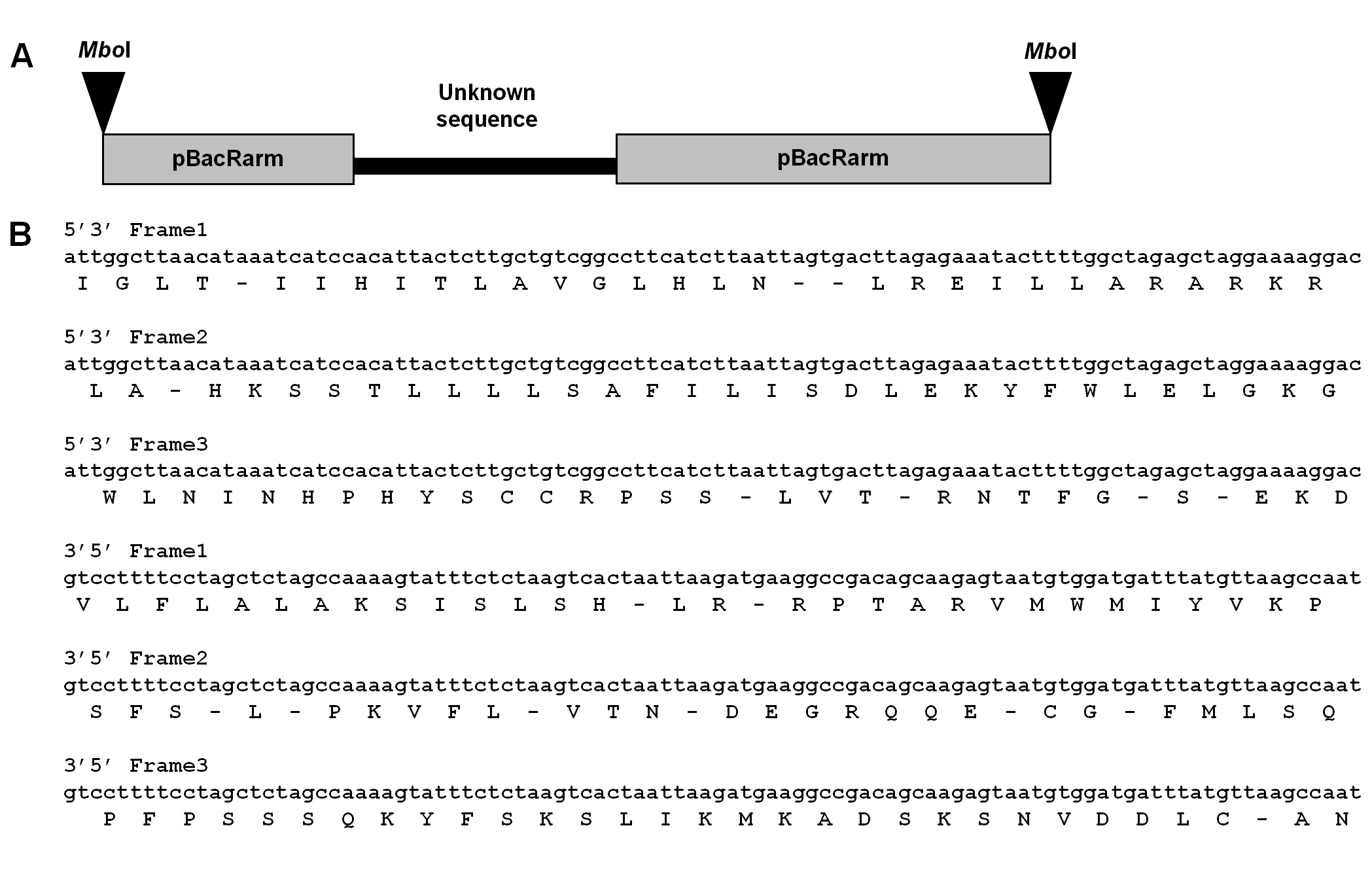

Supplement: Figure S2 — Gene sequence of inverse PCR fragment. A. A schematic of the inverse PCR product sequence. Transgenic genomic DNA was cut with MboI and was self-ligated to form circularized DNA which was used as a template for PCR with pBac-specific primers. As expected, the amplified product (97 bp) from the putative insertion site was flanked with known pBac sequence. B. Putative insertion site sequence and translation in all 6 frames. Translation is presented using the one-letter symbol for each amino acid. Stop codons are represented using a dash (-). (0.16 MB TIF) [file ppat.1001003.s003.tif]

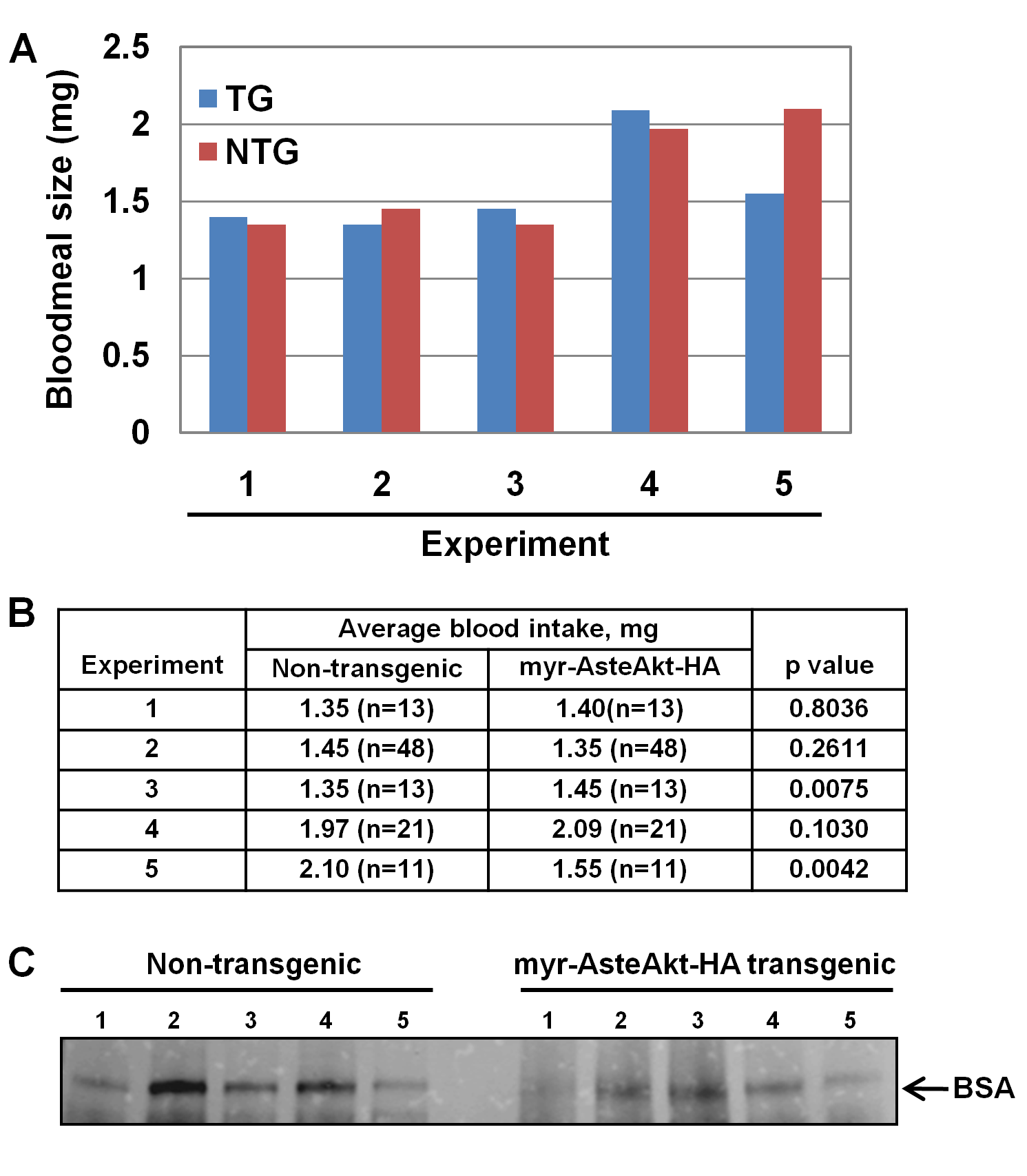

Supplement: Figure S3 — Bloodmeal ingestion and digestion are not affected in myr-AsteAKT-HA transgenics. A and B. Transgenic myr-AsteAKT-HA females ingested the same amount of blood as non-transgenic siblings. Average blood intake was calculated as a difference between an average weight of engorged females in the given weight group before and after bloodfeeding. The p-values reflect difference in the weight between groups of non-transgenic and transgenic mosquitoes within one weight category after a bloodmeal. C. Immunoblot analysis of midguts from five individual myr-AsteAKT-HA transgenic and non-transgenic females 24 h after feeding on bovine blood did not detect obvious differences in the amount of full length BSA remaining in the gut. Each lane was loaded with 0.1 midgut equivalent and probed with an anti-BSA antibody. (0.19 MB TIF) [file ppat.1001003.s004.tif]
